# Oral administration of amphotericin B nanoparticles: antifungal activity, bioavailability and toxicity in rats 

**DOI:** 10.1080/10717544.2016.1228715

**Published:** 2017-02-03

**Authors:** Mahasen A. Radwan, Bushra T. AlQuadeib, Lidija Šiller, Matthew C. Wright, Benjamin Horrocks

**Affiliations:** 1 Department of Pharmaceutical Practice, College of Pharmacy, Princess Nourah bint Abdelrahman University, Riyadh, Saudi Arabia,; 2 Department of Pharmaceutics and Pharmaceutical Technology, College of Pharmacy, Egyptian Russian University, Bader City, Egypt,; 3 Department of Pharmaceutics, College of Pharmacy, King Saud University, Riyadh, Saudi Arabia,; 4 School of Chemical Engineering and Advanced Materials, Herschel Building, Newcastle University, Newcastle upon Tyne, UK, and; 5 Institute of Cellular Medicine, Leech Building, Medical School, Newcastle University, Newcastle upon Tyne, UK

**Keywords:** Amphotericin B, oral delivery, nanoparticles, bioavailability, PLGA-PEG, efficacy, nephrotoxicity

## Abstract

Amphotericin B (AMB) is used most commonly in severe systemic life-threatening fungal infections. There is currently an unmet need for an efficacious (AMB) formulation amenable to oral administration with better bioavailability and lower nephrotoxicity. Novel PEGylated polylactic-polyglycolic acid copolymer (PLGA-PEG) nanoparticles (NPs) formulations of AMB were therefore studied for their ability to kill *Candida albicans* (*C. albicans*). The antifungal activity of AMB formulations was assessed in *C. albicans*. Its bioavalability was investigated in nine groups of rats (*n* = 6). Toxicity was examined by an *in vitro* blood hemolysis assay, and *in vivo* nephrotoxicity after single and multiple dosing for a week by blood urea nitrogen (BUN) and plasma creatinine (PCr) measurements. The MIC of AMB loaded to PLGA-PEG NPs against *C. albicans* was reduced two to threefold compared with free AMB. Novel oral AMB delivery loaded to PLGA-PEG NPs was markedly systemically available compared to Fungizone® in rats. The addition of 2% of GA to the AMB formulation significantly (*p* < 0.05) improved the bioavailability from 1.5 to 10.5% and the relative bioavailability was > 790% that of Fungizone®. The novel AMB formulations showed minimal toxicity and better efficacy compared to Fungizone®. No nephrotoxicity in rats was detected after a week of multiple dosing of AMB NPs based on BUN and PCr, which remained at normal levels. An oral delivery system of AMB-loaded to PLGA-PEG NPs with better efficacy and minimal toxicity was formulated. The addition of glycyrrhizic acid (GA) to AMB NPs formulation resulted in a significant oral absorption and improved bioavailability in rats.

## Introduction

Amphotericin B (AMB) has been the gold standard treatment for severe systemic life-threatening fungal infections since 1959 (Bekersky et al., 1999; Cifani et al., 2012). It has been used as the first-line treatment for visceral leishmaniasis, a life-threatening parasitic disease, in the endemic area of Bihar, India (Sundar et al., 2010). Unfortunately, AMB formulations are only available for parenteral administration.

The amphipathic nature of AMB significantly reduces its solubility in water and most organic solvents. Its aqueous solubility is improved by adding sodium deoxycholate to produce a colloidal dispersion after reconstitution for intermittent intravenous (iv) infusion (Fungizone®). However, severe side effects are associated with the administration of Fungizone®. Nephrotoxicity is the most serious chronic adverse effect of AMB; the serum creatinine concentration (Scr) increases in > 80% of patients receiving the drug (Sachs-Barrable et al., 2008; Tonomura et al., 2009; Chuealee et al., 2011). Additionally, AMB could induce hematological side-effects (Brajtburg & Bolard, 1996; Yu et al., 1998; Adams & Kwon, 2003).

There is limited information regarding AMB metabolism and tissue distribution (Egger et al., 2001). The primary route of its elimination is not known (Drew, 2013). AMB state (monomers or aggregates) affects its efficacy and toxicity. Nishi and his coinvestigators have suggested that AMB is therapeutically active in its monomeric forms while the existence of aggregates forms are responsible for its toxicity (Nishi et al., 2007).

The development of parenteral AMB lipid-based formulations, Abelcet®, Ambisome® and Amphocil®, which have shorter course of therapy (3–5 days), are effective and exhibit lower toxicity when compared to Fungizone® (Torrado et al., 2008; Sundar et al., 2010). However, their cost has restricted widespread use (Sachs-Barrable et al., 2008; Falamarzian & Lavasanifar, 2010).

AMB is also characterized by instability at gastric pH and is unable to cross the mucosal barrier of the GI tract and enter the blood stream. There has been some effort to formulate AMB for oral administration. These include formulating AMB as nanosuspensions (Kayser et al., 2003), as Poly(lactide-co-glycolide) nanoparticles (NPs) employing vitamin E-TPGS as a stabilizer (Italia et al., 2009; Italia et al., 2011), as lipid-based oral formulation using Peceol (Sachs-Barrable et al., 2008) or as liquid antisolvent precipitation NPs (Zu et al., 2014). Furthermore, AMB has been loaded to Peceol and PEG-phospholipids (iCo-009) (Gershkovich et al., 2010; Sivak et al., 2011), to carbon nanotubes (Prajapati et al., 2012), to gelatin-coated lipid NPs (Jain et al., 2012), to Chitosan–EDTA conjugates (Singh et al., 2013) and to Cubosomes (Yang et al., 2012; Yang et al., 2014). The most recent reports include AMB liposomes containing ceramides (Skiba-Lahiani et al., 2015) and AMB encapsulated with a chitosan derivative (Serrano et al., 2015). These oral drug deliveries were developed to enhance the solubility and gastrointestinal permeability of AMB. In most cases, these formulations failed to increase the absorption of orally administered AMB and none of them has been introduced to the market (Ibrahim et al., 2012; Yang et al., 2012).

Glycyrrhizic acid (GA) is a major constituent of licorice, a triterpene glycoside, with steroid-like, antiallergic and antiviral activities (Pompei et al., 1980). It has been used orally as a sweetener and component of oriental medicines (Imai et al., 1999; Anand et al., 2010). In the field of drug delivery, GA possesses *in vivo* enhancing activity with respect to the oral absorption of peptides such as calcitonin (Imai et al., 1999). It is reported as non-toxic oral absorption enhancer to improve the oral bioavailability of different drugs (Radwan & Aboul-Enein, 2002; Chen et al., 2009; Yang et al., 2015).

We hypothesized that loading AMB to PEGylated polylactic–polyglycolic acid copolymer (PLGA–PEG) NPs would improve AMB solubility; decrease its toxicity (since the drug release would be controlled from this delivery system) and AMB aggregation (thereby further decreasing its toxicity toward mammalian cells) while maintaining it in a monomeric form that favors antifungal activity (Brajtburg & Bolard, 1996; Torrado et al., 2008).

Two reports on the use of PLGA–PEG NPs as AMB parenteral delivery systems has recently published. The first study was concern about delivering AMB as a mannose-anchored engineered nanoparticulate for macrophage targeting (Nahar & Jain, 2009), while the second paper encapsulated AMB in PLGA-PEG NPs to increase AMB solubility and to target the macrophages of infected tissues during visceral leishmaniasis (Kumar et al., 2015). To our knowledge, no published data about the development of AMB loaded to PLGA-PEG NPs for oral AMB delivery other than by Al-Quadeib et al. (2015); the *in vitro* studies of the developed formulations indicated promising oral drug delivery system with acceptable drug content and dissolution rate. The aim of this study was to examine the practicability, efficacy, oral bioavailability and safety of these novel AMB formulations in rats. The *in vitro* AMB antifungal activity on *Candida albicans* (*C. albicans*) of these formulations was initially evaluated. Pharmacokinetics investigation of formulations after iv and oral administrations to rats and the feasibility of GA as an absorption enhancer to improve AMB bioavailability was then investigated. Toxicity was examined via an *in vitro* blood hemolysis test and via blood urea nitrogen (BUN) and plasma creatinine (PCr), as indicators of nephrotoxicity after single and multiple dosing of AMB in rats.

## Materials and methods

### Materials

AMB (99.8% purity), clopidogrel, the internal standard (IS), GA and formic acid were purchased from Sigma–Aldrich (St. Louis, MO).

PEGylated Poly (D,L-lactide-co-glycolide) copolymer: RGPd 50155 Diblock with molecular weight 6000 Da (Lactic to glycolic acid molar ratio of 1:1) with 15% polyethylene glycol and Poly(D,L-lactide): Poly(D,L-lactide) named R 203 H Monoblock were supplied by Boehringer Ingelheim (Ingelheim, Germany). Fungizone® (AMB micelle dispersion) was obtained from Bristol-Myers Squibb (Montreal, Canada). RPMI 1640 medium was purchased from Gibco/BRL (Grand Island, NY). Sabouraud Dextrose Agar plates (RODAC™) were obtained from BD Diagnostics (Becton, Dickinson and Company, NJ). All other reagents and chemicals were of HPLC analytical grade, and were used as received. Water was deionized and purified by a Milli-Q Reagent Grade water system (Millipore Corporation, Bedford, MA).

### Preparation of AMB-loaded PLGA-PEG copolymer

AMB loaded to PLGA-PEG was formulated according to Al-Quadeib et al (2015) with modification through addition of GA during formulation or prior to administration as shown below. Table 1 shows the criteria of the selected AmB NPs, from previous study; having a narrow size distribution with smallest possible mean particle size, highest drug encapsulation efficacy and drug release within 24 h. Physicochemical characterizations (particle size analysis, FTIR, DSC and dissolutions were done in our previously published work (Al-Quadeib et al., 2015).

**Table 1.** The characteristics of the selected formulations.

Parameter Formulation	Polymer	Drug content, mg/batch	Mean particle size, nm	Drug encapsulation efficacy, %	Drug release within 24 h, %
F1	PLGA-PEG	20	23.8 ± 4.8	48.3 ± 4.2	61.2 ± 3.2
F2	PLGA-PEG	40	25.3 ± 2.7	56.5 ± 3.9	59.4 ± 5.7
F3	PLA	40	539.9 ± 51.1	27.2 ± 3.2	42.8 ± 2.6

AMB loaded to PLGA–PEG NPs formulation (C6) was selected as the base formulation for this study and is named F1. When GA was added to F1 just prior to administration the formulation is called F1-GA-out-1 and F1-GA-out-2, where, 1 and 2 refer to the percentage of GA added. When GA 2% (w/v) was added to the organic phase during F1 preparation, the resulted formulation is called F1-GA-in-2.

F2 was prepared by doubling the amount of AMB in the formulation C6. When GA was added during preparation, the formulation is called F2-GA-in-2, while F2-GAout-2 is the same composition but the GA was added just prior to administration. To investigate the feasibility of PLGA-PEG NPs versus non-PEGylated polymer, PLA, AMB loaded to PLA NPs formulation (F3) was prepared by the same technique of C7. All formulations were prepared at least in triplicate.

### *In vitro* antifungal activity

*Candida albicans* (ATCC 90028) (*C. albicans*) was used for testing the efficacy of AMB *in vitro*. The minimum inhibitory concentrations (MICs) of AMB in Fungizone®, F1 and F2 were determined by broth dilution according to the National Committee for Clinical Laboratory Standard “NCCLS document M27-A, (Standards, 2002)”. Briefly, *C. albicans* cell suspensions of ∼1 × 10^6^ cells/ml were diluted 1:50 in RPMI-1640 growth medium and 100 μl dispensed into a microliter tray containing a serial concentration of AMB 0.05–1.5 μg/ml. A solution of 5 mg/ml was prepared in DMSO for free AMB and in water for Fungizone®, F1 and F2 immediately before use. The tray was incubated for 24 and 48 h at 37 °C. The yeast were grown on Sabouraud Dextrose Agar (SDA) plates and inoculated into RPMI 1640 broth medium to yield a final inoculum concentration of 10^4^ yeast cells/ml (checked by doing a viable colony count on SDA plates). Two wells containing drug-free medium and inoculum were used as control. The inoculated plates were incubated at 35 °C for 24 h. The growth in each well was estimated visually. The MIC was defined as the lowest drug concentration (MIC) that resulted in complete inhibition of visible growth. The MIC was recorded to be the lowest concentration of the AMB that prevented visible growth of *C. albicans* and expressed in μg/ml. The end-point was determined as the concentration to produce optically clear wells (MIC-0).

### Dosing of animals and blood sampling

All the experiments were performed in accordance with the ethical guidelines established and approved by the committee on the use and care of laboratory animals at King Saud University. Animals were provided from the animal house of King Saud University. The rats were housed in polypropylene cages in an animal facility with a 12 h light-dark cycle and controlled temperature and humidity. Rats were given free access to water *ad libitum* and fasten for 10 h before the study. Standard rat chow was given after 2 h of dosing for the duration of the study.

Table 2 shows the composition and method of *in vivo* administration of the formulations tested in rats.

**Table 2.** The composition, dose and route of administration of the selected formulations.

Animal group	Route of administration	Formulation	Carrier/polymer	Drug amount, mg	GA % IN	GA % OUT	Dose mg/kg
I	PO	Fungizone®	Sodium deoxycholate	50	–	–	10
II	iv	F1	PLGA–PEG	20	–	–	1
III	PO	F1		20	–	–	10
IV	PO	F1-GA-out-1		20	–	1	10
V	PO	F1-GA-out-2		20	–	2	10
VI	PO	F1-GA-in-2		20	2	–	10
VII	PO	F2-GA-in-2		40	2	–	20
VIII	PO	F2-GA-out-2		40	–	2	20
IX	PO	F3	PLA	40	–	–	20

Immediately before administration, the specific weight of the lyophilized AMB-loaded PLGA–PEG NPs were dispersed in water either alone or in combination with GA as 1 or 2% (w/v) as indicated. The volume of the PO colloidal dispersion dose was 1.0 ml.

The study was conducted into two phases; phase I was intended to examine the bioavailability of AMB in the selected formulations while phase II was designed to investigate the toxicity of AMB in these formulations as shown below.

After animals dosing, blood samples (600 μl) were withdrawn from the venous-orbital plexus from each rat in heparinized tubes. Carbon dioxide was used to slightly anesthetize the animals during blood sampling. Plasma samples were separated by centrifugation at 4000 rpm for 15 min and were stored at −20 °C prior to drug assay.

### Bioavailability study

Fifty-four albino Sprague-Dawley male rats (353.2 ± 26.6 g) were randomly divided into nine groups (I–IX, *n* = 6) and were dosed according to Table 2. Blood samples from the same rats were collected at 0, 1, 3, 6 and 0.5, 2, 4, 8, 24 h after drug administration in two different occasions, separated by 3 weeks.

### Nephrotoxicity and histopathological studies

The selected formulations of AMB loaded to PLGA-PEG NPs for the *in vitro* efficacy and *in vitro/in vivo* toxicity investigations were F1 and F2, which contain 20 and 40 mg of AMB, respectively, without any addition of GA.

Fifteen albino Sprague-Dawley rats with 200–300 g weight were randomly divided into five groups (1–5), (*n* = 3). Group-1 was kept as control without any treatment; group-2 received iv blank NPs with no AMB; group 3 was given Fungizone® (1.0 mg/kg) as a slow iv injection in the tail vein; group-4 received iv administration of F1 (1.0 mg/kg) while group-5 received iv administration of F2 (1.0 mg/kg).

After 24 h post first dose, blood samples were collected from each rat as the single dose samples (SD) experiment. The animals were received daily the same treatment for 7-days and 24 h post-the 7th dose, blood samples were collected for the multiple dose (MD) experiment.

BUN and PCr were measured using an automatic analyzer 7180 (Hitachi High- Technologies Co., Tokyo, Japan) after the single and multiple dosing administrations.

For histopathological analysis, one kidney from each rat was fixed in neutral buffered 10% formalin for ≥48 h, bisected and embedded in paraffin. Sections of 5.0 μm thickness were cut from each kidney, and stained with hematoxylin and eosin. For histopathological analysis of liver, a piece of median lobe from the livers were removed from each rats and placed immediately in 10% neutral buffered formalin. Sections of 5.0 μm thickness were cut and stained with hematoxylin and eosin similarly to kidney tissue.

### *In vitro* hemolytic activity of AMB

*In vitro* hemolytic activity of AMB-loaded to PLGA-PEG NP of the selected formulations (F1 and F2) were assessed using isolated rat red blood cells (RBCs) according to Jain and Kumar (Jain & Kumar, 2010). Briefly, blood samples from healthy Sprague-Dawley male rats (250–350 g) were collected by cardiac puncture under anesthesia directly into heparinized blood collecting vials. The RBCs were separated by centrifuging the whole blood at 3000 rpm for 15 min, the supernatant along with buffy coat were pipetted off and discarded. RBCs were washed thrice with 0.15 M isotonic phosphate buffer saline (PBS) of pH 7.4 and was dispersed in PBS to obtain 1% hematocrit. RBCs were used on the same day for further experiments. Subsequently, 1.0 ml of the RBCs suspension was mixed with 1.0 ml of PBS containing 20, 50 or 100 μg/ml AMB equivalent formulations (Fungizone®, F1 or F2) and was incubated at 37 °C in a shaking water bath at 100 rpm. The experiment was performed in triplicate. After 8 and 24 h of incubation, any hemolysis was stopped by reducing the temperature to 0° C and un-lysed RBCs were removed by centrifugation for 10 min at 3000 rpm. The supernatant was collected and the erythrocyte pellet was lysed with sterile distilled water and analyzed for the extent intact RBCs using a spectrophotometer set at the absorption maximum of hemoglobin (540 nm). Control RBC (2 × 10^8^ cells/ml) incubated with PBS alone was used to estimate the total hemoglobin content.

Incubation of RBCs with distilled water (as positive control) was considered to cause 100% hemolysis. Results were expressed as a percentage of hemolysis as given in the following equation:

$$\mathrm{Hemolysis}\%\;=\;\left[\left({\mathrm{Abs}}_{S}-\;{\mathrm{Abs}}_{0}\right)/\left({\mathrm{Abs}}_{100}-\;{\mathrm{Abs}}_{0}\right)\right]\times \;100$$

Where, *Abs*_S_ is the absorbance of the sample, *Abs*_0_ is the average absorbance of the buffer; negative control, and *Abs*_100_ is the average absorbance of the lysed samples (in purified water; positive control). The remaining hemoglobin was calculated as a percentage of the total content. Results are given as the mean of one experiment representative of three experiments carried out with each concentration in triplicate.

### Chromatographic conditions

Analysis was carried out on a Waters Acquity UPLC™ system (Waters, Milford, MA). The analytical method used was adapted from Al‐Quadeib et al. (2014) using rat plasma instead of human plasma. There was no significant difference (*p* > 0.05) between the standard curves best fit equations on slopes, intercepts and correlation of the human and rat plasma. The precision and accuracy of the developed LC MS/MS method were measured for the concentration range of 100–4000 ng/ml and was shown no significant difference among inter- and-intra-day analysis (*p* > 0.05) in rat plasma. Excellent linearity was observed over the investigated range with correlation coefficient, *r* > 0.995 (*n* = 6/day). The assay was able to detect AMB concentrations for all time points after iv and PO administrations to rats without any modification.

### Pharmacokinetics and statistical analysis

All data were expressed as the mean ± SD, if not specified otherwise, of six replicates for the assay or the rats study. The standard curves were calculated by linear regression without weighting, using the equation *Y* = 0.0007*X* – 0.127, where *Y* is the AUP ratio of the drug to the IS, −0.127 is the intercept, 0.0007 is the slope, and *X* is AMB concentration. The relative standard deviation (RSD) was calculated for all values.

The plasma AMB concentration versus time data of the rats were analyzed using a model-independent method for the main purpose of this study (Gibaldi & Perrier, 1982). The mean maximum concentration (*C*_max_) and the time to reach *C*_max_ (*T*_max_) were derived directly from the individual plasma levels. The elimination rate constant (*k*) was calculated from the slope of the regression line that best fit the terminal part (last three to four points) of the log–linear concentration–time profile of AMB. The terminal half-life (*t*_1/2_) was calculated from 0.693/*k*. The area under the curve from time 0 to 24 h (AUC0–24) was estimated by linear trapezoidal rule and was extrapolated to time infinity (AUC) by the addition of *C*_n_/*k* where, *C*_n_ is concentration of the last measured plasma sample. The total body clearance (Cl) was estimated using the equation Cl = dose/AUC. The volume of distribution (V) was determined from the equation *k* = Cl/*V*. For oral data, the absorption rate constant *k*_a_ was estimated by the method of residual (Tozer & Rowland, 2006). The absolute bioavailability (F) was calculated by the equation (AUC_po_/Dose_po_)/(AUC_iv_/Dose_iv_) after iv of F1 and oral administration of the tested formulations listed in Table 2 while the relative bioavailability (*F*_rel_) was calculated using (AUC, PO formulation/AUC, F1-PO) *F*_rel_ to Fungizone (AUC, PO formulation/AUC, Fungizone-PO).

All statistical differences in data were evaluated using IBM SPSS Statistics 21 (IBM cooperation, New York, NY). The Student *t*-test was used to examine the concentration difference at each day and one-way analysis of variance (ANOVA) was employed to assess the reproducibility of the assay and other tests were used when needed. *p* < 0.05 was considered significant.

## Results

### Bioavailability of amphotericin B in rats

**Figure 1.**
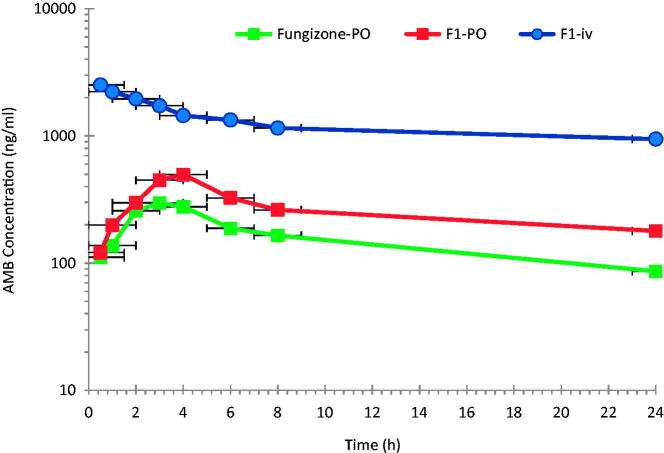
Mean plasma AMB concentration–time profiles after intravenous administration of 1.0 mg/kg of F1-iv and oral administrations of 10 mg/kg of F1-PO and Fungizone**®** to rats (*n* = 6).

Novel oral biodegradable stealth polymeric nanoparticles of AMB have been successfully fabricated by a modified emulsification–diffusion technique using PLGA–PEG (Al-Quadeib et al., 2015). Figure 1 shows the logarithmic mean plasma concentration versus time profiles of AMB following F1 formulation as a 1.0 mg/kg single dose (F1-iv) and 10 mg/kg (F1-PO) to two different groups of rats via iv and PO administrations, respectively. Fungizone®, 10 mg/kg, (Fungizone®-PO) was given to a third group of rats via PO administration for comparison.

After iv administration, a two compartment open model was considered to adequately describe the kinetics of AMB in rat plasma with a fast distribution, over the first 4 h, followed by slow elimination. The oral administration of AMB as F1-PO or Fungizone® show the same trend, after the absorption phase. However, a model-independent method was used instead for the main purpose of this study and for simplicity (Gibaldi & Perrier, 1982).

The pharmacokinetic parameters of the three AMB dosage forms (F1-iv, F1-PO and Fungizone®-PO) in rats are summarized in Table 3. After iv administration of F1-iv, AMB shows a relatively long elimination *t*_1/2_ ranging from one to two days in rats due to its low Cl (8–23.2 ml/h/kg) and large apparent volume of distribution in the range of 552.7–806.3 ml/kg.

**Table 3.** Pharmacokinetic parameters of AMB after iv and oral administrations of AMB-loaded PLGA-PEG formulations and fungizone in rats (*n* = 6).

Tested parameters	F1-iv	F1-PO	Fungizone®-PO
Dose, mg/kg	1	10	10
*C*_max_, ng/mL	–	480.2 ± 38.7	298.2 ± 28.3
AUC_0–24h_ (μg.h/l)	–	6100 ± 514.9	3720.8 ± 617.5
AUC (μg.h/l)	84211.2 ± 33935	12325.1 ± 1511.3	9832.4 ± 1657.5
k (h^−1^)	0.0208 ± 0.0084	0.0298 ± 0.0060	0.0019 ± 0.0015
Cl (ml/h/kg)	13.8 ± 6.1		
*t*_1/2_ (h)	38.1 ± 14.8	24.0 ± 4.4	35.3 ± 2.6
V (ml/kg)	666.1 ± 109.7		
*F*	–	0.0146	0.0117
*F*_rel Fungizone_	–	1.3	

Although the iv dose was one tenth the PO doses of either F1-PO or Fungizone® PO, F1-iv shows a pronounced higher plasma concentration of AMB (*p* < 0.05). A statistically significant higher *C*_max_ of AMB was observed following F1-PO than following Fungizone®-PO administrations. The observed mean plasma AUC_0–24 h_ and AUC_0–∞_ after F1-PO was about 63.7% and 36.4%, respectively, higher than that of Fungizone®-PO. The bioavailability (*F*) of F1-PO was 36.4% higher than that of Fungizone® PO due to the improvement of AMB release and absorption when loaded to PLGA-PEG but the results were not satisfactory.

**Figure 2.**
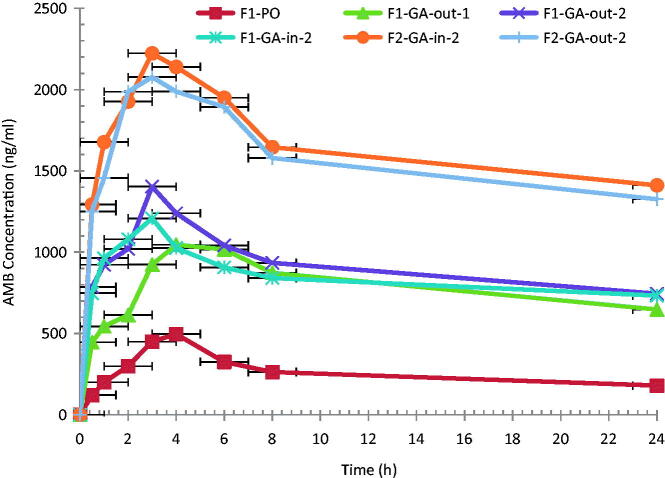
Mean plasma AMB concentration–time profiles following single oral administration of 10.0 mg/kg of AMB-loaded PLGA-PEG formulations in rats (*n* = 6).

To investigate the effect of GA as an absorption enhancer, F1 and F2 were given to rats with GA, added just prior to the administration or were included in the organic phase during AMB loaded to PLGA-PEG NP preparation. AMB mean plasma concentration–time profile after single oral doses of AMB-loaded PLGA–PEG formulations with and without the addition of the absorption enhancer GA are depicted in Figure 2 and the pharmacokinetic parameters are presented in Table 3.

*C*_max_ after F1 without GA was 1.6 times that after Fungizone (*p* < 0.05). A significant increase (*p* < 0.05) in AMB *C*_max_ (2.3-fold) with 4.3-fold increase in AUC (332.3%) was observed after the addition of 1% of GA to the PO formulations, just prior to the administration. This is an indication of the efficiency of GA as an absorption enhancer for AMB.

Increasing the GA content from 1% (F1-GA-out-1) to 2% (F1-GA-out-2), showed a significant increase in *C*_max_ (2.97 fold) and in AUC (390.7%) compared to F1 formulation without GA. Further increase in GA content to 3% showed no significant change in either *C*_max_ or AUC of AMB profile, data not shown. Therefore, GA at 2% was selected as the optimum amount to be added to the tested formulations.

It was noticed that the addition of GA during preparation process (F1-GA-in-2) or just prior to the administration (F1-GA-out-2) showed no significant difference (*p* > 0.05) in the AUC (13.5%), with a 27.9% increase in the *C*_max_ value. This is an indication that the efficacy of GA as an absorption enhancer for AMB is not changed either GA added just prior to administration or during the preparation.

Doubling the amount of the AMB in the formulation from 20 mg (F1) to 40 mg (F2) resulted in doubling the AUC of F1-GA-in-2 and F2-GA-in-2 (20216.2 ± 1593 and 38808.6 ± 4033 μg.h/l, respectively). The same trend was observed when GA was out, therefore, AMB shows linear kinetic within the tested amount in the formulation.

It should be mentioned that for each tested formulation, in rats, the value of the absorption rate constant of AMB loaded to PLGA-PEG NPs, *k*_a_, was much greater than the value of elimination rate constant *k*, which indicates a fast release of AMB with no sign of the flip-flop’ model (Gibaldi & Perrier, 1982). The highest *k*_a_ among the tested formulations was F1-GA-in compare to the others. Therefore, the addition of GA during preparation of F1 resulted in higher release of AMB.

The absolute bioavailability (*F*) of AMB from tested AMB loaded to PLGA-PEG NP formulation was calculated, Table 4. Upon the addition of 2% GA during the formulations, the *F* was improved from 1.5 to 10.5% (sixfolds). The *F*_rel_ was improved from 332 to 616.5% and 25 to 800% after the addition of GA compared to F1-PO and Fungizone, respectively. The highest improvement in F_rel_ was detected in formulation F1-GA-in-2.

**Table 4.** Pharmacokinetic parameters of AMB (mean **±** SD) after a 10 mg/kg single oral administration of AMB as Fungizone**®** and AMB-loaded PLGA-PEG NP formulations in rats (*n* = 6).

Parameters	F1-PO	F1-GA-out-1	F1-GA-out-2	F1-GA-in-2	F2-GA-in-2	F2-GA-out-2	Fungizone®-PO
AUC_0–24h_ (μg.h/l)	6100 ± 514.9	18 818.7 ± 2851	21 803.2 ± 1346	20 216.2 ± 1593	38 808.6 ± 4033	37 364.6 ± 2883	3720.8 ± 617.5
AUC (μg.h/l)	12325.1 ± 1511	53 287.0 ± 24 997	60 478.8 ± 7437	88 307.9 ± 36092	12 0689.8 ± 30 628	10 8366.9 ± 19 736	9836.3 ± 1654
*C*_max_ (ng/ml)	480.2 ± 38.7	1115.0 ± 172	1426.3 ± 133	1239.4 ± 75	2387.2 ± 294	2147.2 ± 176	298.2 ± 28
*k*_a_,/*h*	0.994 ± 0.196	0.72 ± 0.35	1.244 ± 0.513	1.684 ± 1.374	1.68 ± 1.15	1.35 ± 0.38	0.904 ± 0.0.72
*t*_1/2_ (h)	24.0 ± 4.4	34.6 ± 15.1	36.0 ± 5.5	42.7 ± 4.2	41.3 ± 11.5	37.1 ± 6.5	35.3 ± 2.6
Mean *F*	0.015	0.063	0.072	0.105	0.072	0.064	0.0117
Mean *F*_rel,_ *_F_*_1_	–	4.3	4.9	7.2	4.9	4.4	0.78
Mean *F*_rel, Fungizone_	1.3	5.4	6.1	9.0	6.1	5.5	–

Although the iv dose was one tenth the PO doses of either F1-PO or Fungizone®-PO, F1-iv shows a pronounced higher plasma concentration of AMB (*p* < 0.05). This could be attributed to either incomplete absorption of AMB from these formulations or because the drug was subjected to a significant first pass metabolism in the liver after absorption from the GI tract.

Moreover, loading AMB to non-PEGlyated polymer (poly (D, L-lactide-co-glycolide, RG 502 H), showed no absorption of AMB at all the time points. This is a confirmation of the importance of the PEG in PLGA polymer to promote the absorption of AMB after PO administration.

### *In vivo* nephrotoxicity

Nephrotoxicity is the most serious adverse effect of AMB after chronic exposure; the Scr increases in more than 80% of patients receiving this drug (Sabra and Branch, 1990; Tonomura et al., 2009). In the conventional solution formulation, the drug is present as micelles. As soon as the drug solution enters the bloodstream it separates from the micelles and this appears to be critical to subsequent nephrotoxic effects. The incidence of AMB nephrotoxicity is very high, varying in studies between 49% and 65% (Deray, 2002). Nephrotoxicity is defined in most studies as a doubling of baseline creatinine (Cr) levels (100% increase from the serum baseline) or greater than 2.5 mg/dl in human (Miller et al., 2004).

**Figure 3.**
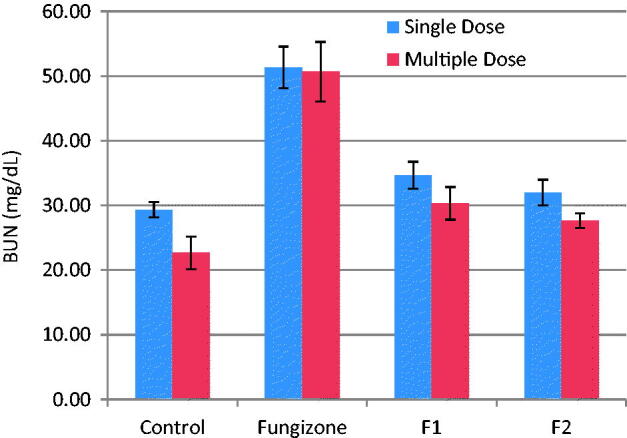
BUN, mg/dL, (mean ± SD) after single and multiple iv doses (5 mg/kg) of AMB and AMB loaded to PLGA-PEG NP to rats (*n* = 3).

**Figure 4.**
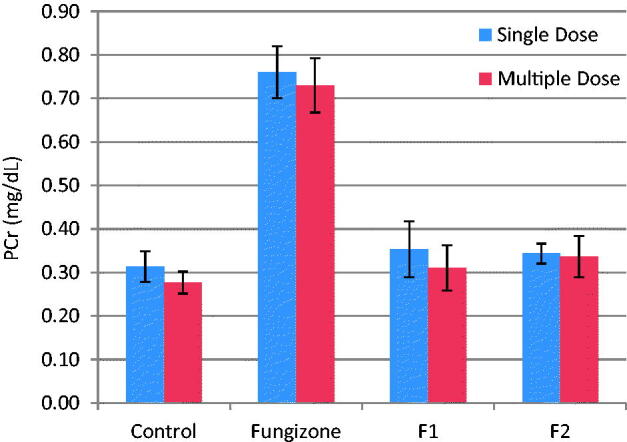
Plasma Cr, mg/dL, (mean ± SD) after single and multiple iv doses (5 mg/kg) of AMB and AMB loaded to PLGA-PEG NP to rats (*n* = 3).

The present investigation examined two biomarkers (BUN and PCr) of renal injury. No mortality was observed after iv doses in any of the rats during the study. Relevant changes in serum chemistry are summarized in Figures 3 and 4. In the rats treated with iv Fungizone®, there was a significant (*p* = 0.003 and *p* = 0.001) increase in BUN and PCr levels, respectively, as compared to control rats, indicating that Fungizone administration resulted in nephrotoxicity upon administration as a single or multiple doses. However, iv administration of AMB-loaded to PLGA-PEG NPs led to non-significant difference (*p* > 0.05) in BUN and PCr levels in the rats, as compared to control rats, indicating lower nephrotoxicity by AMB when loaded to PLGA-PEG NPs as compared to Fungizone®.

PCr were in the range of 0.31–0.34 mg/dl for both formulations, which is within the normal range for rat plasma. Therefore, AMB loaded to PLGA-PEG NPs, showed minimal renal effects than Fungizone® over the short duration of treatment.

**Figure 5.**
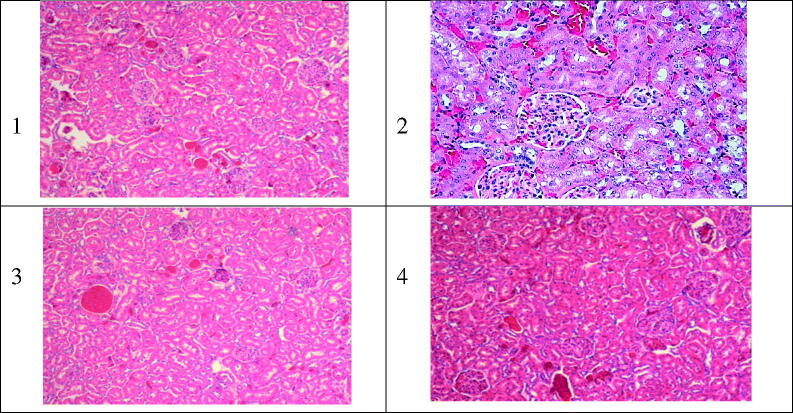
Typical kidney tissue alterations verified in rats treated with AMB or its equivalent dose as 1.0 mg/kg of body weight as iv administration of different Amb-PLGAPEG copolymer. 1) normal kidney tissue; 2,3,4) Fungizone® f1 and f2, respectively, varying degree of nephrotoxicity necrosis related to iv administration of iv doses (5 mg/kg).

The histopathological analysis of kidney tissue after iv administrations of the NPs formulations of AMB and Fungizone®, did not confirm any unusual sign of necrosis in all treated groups except in the Fungizone® treated group. It showed a distinctive necrosis of various degree as presented in Figure 5, which has been approved by the PCr results in the same study. As a conclusion, all the iv administrations of the developed AMB-loaded to PLGA–PEG Diblock copolymer investigated, showed minimal renal damage compared to the reference formulation Fungizone®, over a short duration of treatment.

**Figure 6.**
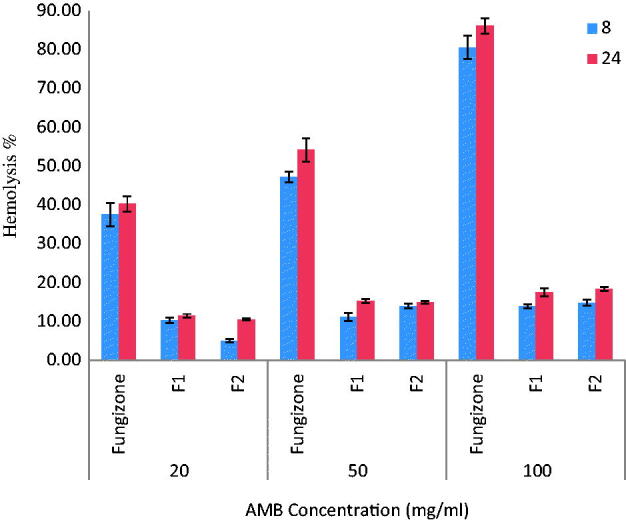
*In vitro* mean RBCs hemolysis following incubation of RBC with Fungizone**®** and different AMB-NPs formulations at concentrations of 20, 50 and 100 μg/ml (*n* = 3).

### *In vitro* hemolysis test

In order to determine the effect of the NPs of AMB on hemolytic profile, the RBS lysis induced by AMB NP selected formulations were compared with the Fungizone®.

The extent of hemolysis induced after incubation of RBCs with F1 and F2 formulations of AMB-NP in comparison with Fungizone® is depicted in Figure 5. The hemolysis was high (84%) in the case of Fungizone® at 100 μg/ml and 43% at 20 μg/ml. Therefore, it seems that Fungizone® is likely to be toxic, even at the lowest concentration used in the experiment. At a similar concentration AMB loaded to PLGA–PEG NPs formulations showed negligible hemolysis. The results in Figure 6 also indicate that the hemolysis was dose-dependent. Regarding the degree of hemolysis, the tested formulations were classified as low hemolytic toxicity 4 to 8 times hemolysis reduction in comparison with Fungizone®, F2 formulation showed the lowest hemolysis reflects a better control over the rate of AMB diffusion from these selective formulations over the Fungizone®. After 24 h incubation, similar results were obtained as that after 8 h incubation, indicating that the releases of AMB from these formulations is slow and it seems that PLGA-PEG NP imparts a protective effect to AMB in preventing the RBC lysis of these formulations.

### *In vitro* antifungal activity

Table 5 shows the antimicrobial activity of pure AMB, Fungizone® and the selected formulations of AMB loaded to PLGA-PEG NP in *C. albicans* after 24 and 48 h incubations. The MIC-0 for pure AMB was found to be 0.5 μg/ml after 24 h and 1.0 μg/ml after 48 h incubation. Similar results are obtained by others (Nishi et al., 2007). Meanwhile, the MIC-0 for AMB loaded to PLGA–PEG NP formulations was reduced by ≥ fourfold.

**Table 5.** Checkboard assay of AMB against *C. albicans* (*n* = 6).

Tested form	MIC-0 (μg/ml) after 24 h	MIC-0 (μg/ml) after 48 h
AMB	0.5 ± 0.01	1.0 ± 0.01
Fungizone®	0.5 ± 0.01	1.0 ± 0.01
*F*1	0.125 ± 0.01	0.125 ± 0.01
*F*2	0.125 ± 0.01	0.125 ± 0.01

## Discussion

Several reports show that parenteral administrations of AMB polymeric nanoparticles formulations, have antifungal efficacy and lower toxicity with increase accessibility of the drug to organs and targeted tissue (Amaral et al., 2009; Laniado-Laborín & Cabrales-Vargas, 2009; Souza et al., 2015). It was also reported that some carriers added during the formulation process affect the aggregation state and hence AMB activity (Legrand et al., 1992). Barwicz et al. have demonstrated the ability of surfactants to reduce the toxicity of AMB via decreasing the aggregation state of the drug (Barwicz et al., 1992; Adams and Kwon, 2003).

Currently, PLAG and PLGA-PEG are being used to improve the release rate of different classes of drugs from NPs systems intended for both parenteral (Allémann et al., 1998; Huh et al., 2003; Packhaeuser et al., 2004; Cheng et al., 2007; Saadati & Dadashzadeh, 2014; Teekamp et al., 2015) and oral administrations (Yoo & Park, 2001; Garinot et al., 2007; Fernandez-Carballido et al., 2008; Khalil et al., 2013; Yan et al., 2015). The ability of PLGA–PEG to enhance bioavailability of hydrophobic drugs, has been demonstrated for different classes of drugs. Curcumin potential efficacy is limited by its lack of solubility in aqueous solvents and poor oral bioavailability. PLGA–PEG NPs were able to increase curcumin bioavailability by 3.5-fold compared to curcumin loaded PLGA NPs alone (Anand et al., 2010).

GA and its derivatives have been utilized in enhancing drug absorption (Tanaka et al., 1992; Imai et al., 1999; Radwan & Aboul-Enein, 2002; Cho et al., 2004; Anand et al., 2010). Significant enhanced rectal absorption of AMB suppositories in rabbits following GA addition in comparison to formulation containing no GA (Tanaka et al., 1992; Anand et al., 2010). Insulin nasal spray formulations for nasal delivery containing GA showed improved bioavailability (Khalil et al., 2013). Additionally oral delivery of heparin is achieved by addition of GA (Motlekar et al., 2006).

Comparing the relative bioavailability of each tested formulation to F1-PO without GA, resulted in a maximum improvement of 798% in *F*_rel_ to Fungizone, which is better than the outcome of the published investigations as presented in Table 6. Sachs-Barrable et al. (2008) was exceptional in improving the relative *F* of AMB but in different system and dose of Fungizone® used.

**Table 6.** Relative bioavailability of AMB in rats, after PO administration of various AMB-loaded NPs in different formulations, in comparison to Fungizone®.

Reference	Formulation/Polymer	Dose of fungizone, mg/kg	AMB Dose in tested formulation, mg/kg	*F*_rel_ to fungizone %
Current study	Copolymer (PLGA-PEG)	10	10	798
(Yang et al., 2012)	Cubosomes	10	10	285
		10	20	702
(Italia et al., 2009)	PLGA	10	10	693
(Sachs-Barrable et al., 2008)	Peceol/AMB	50	50	2197
		50	5	8506

AMB nephrotoxicity is manifested by renal insufficiency due to glomerular and vascular disease in addition to abnormalities in tubular function. Acute AMB-induced nephrotoxicity is characterized by increased BUN and increased PCr levels. BUN is an important biochemical marker, which is routinely evaluated as an indicator of clinical renal function. Abnormally high BUN levels indicate the dysfunction or damage to the kidney. Similarly, PCr is another reliable marker of renal function. Creatinine, the end product of muscle metabolism, is filtered and excreted by the kidneys. Elevated PCr levels is indicative of renal dysfunction (Italia et al., 2009; Tonomura et al., 2009).

The reduced toxicity of AMB-NP observed could be due to the fact that AMB was slowly released from AMB-NP than from the deoxycholate micelles of Fungizone®. AMB is known to induce vasoconstriction, which leads to a decrease of blood flow in the kidney. The observed reduction in the PCr is believed to be caused by a decrease in blood pressure that is associated with a decrease in blood flow in the glomerulus (Tasset et al., 1992; Tiyaboonchai et al., 2001; Risovic et al., 2003; Nahar et al., 2008; Italia et al., 2009; Tonomura et al., 2009; Belkherroubi-Sari et al., 2011).

It is reported that AMB is highly toxic in its aggregated state than in its monomer form (Brajtburg & Bolard, 1996; Nishi et al., 2007). In solution, AMB exists in three different forms; monomers, oligomers and aggregates. The soluble form of AMB exists in monomeric form (Brajtburg & Bolard, 1996; Nishi et al., 2007).

The ratio of absorbance at 348 nm to 409 nm is reported to give the extent of aggregation in AMB (Legrand et al., 1992). Barwicz et al. (1992) report the ratio to be 2 for aggregated species. Most of the AMB formulations have greater A348/A409 values. For instance, Fungizone® has a value of 2.9 while Ambisome® has a value of 4.8 (Mullen et al., 1997). For AMB-loaded PLGA-PEG, the value obtained was less than1.14 showing that AMB was not aggregated in the novel oral formulation. Therefore, assessment of hemolytic toxicity seems to be a prerequisite while developing any formulation of AMB.

The lack of hemolysis activity may reflect the release of monomeric AMB from AMB-loaded PLGA-PEG copolymer as opposed to Fungizone®, which release both aggregated and monomeric forms of the drug (Yu et al., 1998; Adams & Kwon, 2003; Brajtburg & Bolard, 1996). Therefore, the low hemolysis of all AMB-NP as shown in Figure 6 might be attributed to the encapsulation of a nanoaggregate form of AMB, and slow diffusion of AMB from the conjugated polymer. Another explanation, is that during formulation, AMB was added in an aqueous polymer phase that was acidified with 5N HCL (pH 3) (far from the p*K*a of the drug; 3.7 and 10), thus precluding self-association or aggregation.

Fungizone® caused more extent of hemolysis because Fungizone® formulation consist of micellar dispersion of AMB with sodium deoxycholate, which act as surfactant and can induce hemolysis itself in addition to the hemolysis caused by AMB. Fungizone® is well-known for causing hemolysis mainly due to active hemolysis by pore formation and changing electrolyte balance in erythrocytes (Yu et al., 1998; Fukui et al., 2003; Bang et al., 2008; Nahar et al., 2008; Italia et al., 2009; Falamarzian & Lavasanifar, 2010; Jain & Kumar, 2010; Shao et al., 2010; Sheikh et al., 2010; Asghari, 2011).

AMB at concentrations > 0.5 to 1-fold the MIC has a fungicidal activity, while AMB at concentrations < 0.5 to 1-fold the MIC has a fungistatic activity, a finding that has been described previously (Tasset et al., 1992; Pfaller & Barry, 1994; Risovic et al., 2003; Belkherroubi-Sari et al., 2011). The MIC of AMB loaded to PLGA-PEG NP against *C. albicans* was reduced two to three-fold compared with free AMB. Therefore, the prepared AMB-NPs formulations have high therapeutic efficacy and are useful for the treatment of fungal infection including candidiasis.

## Conclusion

In conclusion, an innovative AMB oral formulation was developed which would improve the patient adherence due to the potential reduction of AMB adverse side effects especially nephrotoxicity and induction of hemolysis. PEGylated polymer (PLGA-PEG) enhances the oral absorption of AMB compared to Fungizone®. On a cellular level, AMB loaded to PLGA-PEG copolymer showed potent activity against *in vitro C. albicans* with no pathological abnormalities were observed in rats’ kidney tissues. A further study is needed to investigate the *in vivo* efficacy of the developed formulation.
